# Nanoscale chemical and structural investigation of solid solution polyelemental transition metal oxide nanoparticles

**DOI:** 10.1016/j.isci.2023.106032

**Published:** 2023-01-24

**Authors:** Abhijit H. Phakatkar, Tolou Shokuhfar, Reza Shahbazian-Yassar

**Affiliations:** 1 Department of Biomedical Engineering, University of Illinois at Chicago, Chicago, IL, USA; 2 Department of Mechanical and Industrial Engineering, University of Illinois at Chicago, Chicago, IL, USA

**Keywords:** Chemistry, Inorganic materials, Materials science, Materials chemistry, Materials synthesis

## Abstract

Although it has been shown that configurational entropy can improve the structural stability in transition metal oxides (TMOs), little is known about the oxidation state of transition metals under random mixing of alloys. Such information is essential in understanding the chemical reactivity and properties of TMOs stabilized by configurational entropy. Herein, utilizing electron energy loss spectroscopy (EELS) technique in an aberration-corrected scanning transmission electron microscope (STEM), we systematically studied the oxidation state of binary (Mn, Fe)_3_O_4_, ternary (Mn, Fe, Ni)_3_O_4_, and quinary (Mn, Fe, Ni, Cu, Zn)_3_O_4_ solid solution polyelemental transition metal oxides (SSP-TMOs) nanoparticles. Our findings show that the random mixing of multiple elements in the form of solid solution phase not only promotes the entropy stabilization but also results in stable oxidation state in transition metals spanning from binary to quinary transition metal oxide nanoparticles.

## Highlights

• EELS analysis of solid solution polyelemental transition metal oxides (SSP-TMOs)
• Spinel phase (Mn, Fe)_3_O_4_, (Mn, Fe, Ni)_3_O_4_, (Mn, Fe, Ni, Cu, Zn)_3_O_4_ SSP-TMOs
• Stable oxidation state in SSP-TMOs spanning from binary to quinary nanoparticles
• Increased degree of inversion among octahedral and tetrahedral sites in SSP-TMOs

## Graphical abstract

**Figure:**
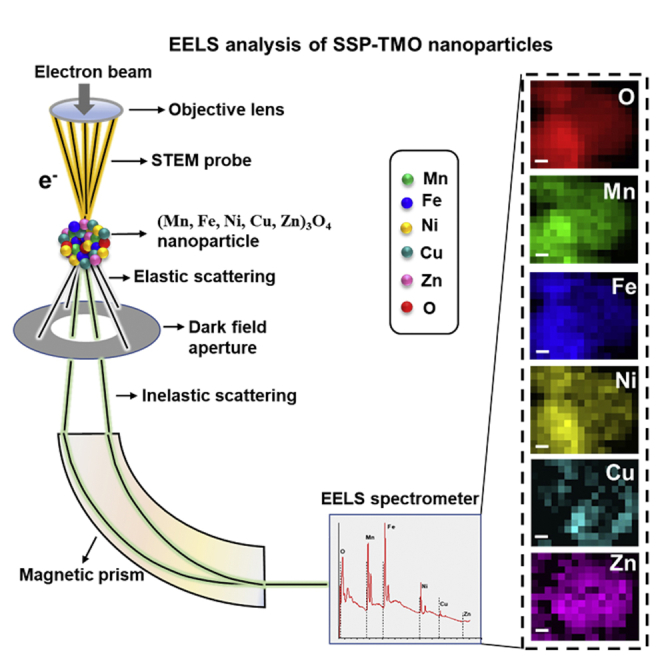

## Introduction

Entropy stabilized transition metal oxides (TMOs) have displayed remarkable potential for the applications in catalysis, energy storage, waste water treatment, and biomedical fields.^1^^,^^2^^,^^3^^,^^4^^,^^5^^,^^6^ The high chemical and structural stability of solid solution polyelemental (SSP)-TMOs is mainly because of the increase in configurational entropy resulting from incorporation of near equimolar ratio of multiple elements.^7^^,^^8^ Jahn-Teller structural distortions resulting from d-orbital splitting at octahedral sites plays an important role on the electronic and structural stability of multi-elemental transition metal oxide nanoparticles.^9^^,^^10^ Yan et al.^11^ showed that copper occupied at octahedral sites (CuO_6_) in the high entropy oxide (HEO) nanoparticles can reduce local structural distortions on application of compressive force (∼40 GPa), allowing the delocalization of the higher energy state (e_g_) electrons into the stable state in the d-orbitals. In addition, the rapid heating and cooling rates promoting the formation of entropy stabilized nanomaterials, facilitate the chemical homogeneity in the high entropy structures by tuning the thermodynamic mixing kinetics leading to the single-phase solid solution emergence.^12^

Electron energy loss spectroscopy (STEM-EELS) is a powerful technique to evaluate the oxidation state of elements at atomic scale. For transition metals, L_2,3_ white lines resulting from the transition of an electron from 2*p* state to 3*d* unoccupied state can give insights on the oxidation state of transition metals.^13^ In fact, the oxidation states with respect to the L_3_ (2p_3/2_) and L_2_ (2p_1/2_) white lines have been studied for SSP-TMOs.^1^^,^^5^^,^^14^^,^^15^ The well-resolved electron energy-loss near edge structures (ELNES) can determine dipole-allowed transitions at higher energy state in the outer shell orbitals.^3^ In addition, EELS is shown to reveal atomic scale hybridization states in the complex perovskite structure by analyzing ELNES at oxygen K-edge.^16^ Recently, EELS technique is utilized for acquiring atomic resolution elemental mapping of HEO nanoparticles for confirming the cation sites occupancies in the crystal structures.^17^^,^^18^ Song et al.^19^ showed utilization of EELS by analyzing oxygen K-edge and L_2,3_ ionization edges for respective transition metals to prove the slower rate of surface oxidation of high entropy alloy nanoparticles under *in-situ* high temperature environmental conditions.

The oxidation states of cations integrated in SSP-TMOs plays decisive role in attaining the microstructural and electronic stability.^20^ Moreover, higher number of oxygen vacancy concentrations in the SSP-TMO structures can promote the low-oxidation state cations.^21^ The alterations caused by the lattice defect sites and the varying cation oxidation states affect the electronic stability of the SSP-TMO nanomaterials.^22^ Such studies indicate that there exists a knowledge gap in the comprehension of the metal cations valance state in the randomly mixing state.

In the present study, for the very first time, we investigated the chemical oxidation states of randomly mixed SSP-TMOs spanning from binary (Fe, Mn)_3_O_4_, ternary (Mn, Fe, Ni)_3_O_4_, and quinary (Mn, Fe, Ni, Cu, Zn)_3_O_4_ nanoparticles by means of EELS in an aberration-corrected STEM. The spinel phase crystal structure of synthesized SSP-TMOs nanoparticles was evaluated using selected area electron diffraction (SAED) and high angle annular dark field (HAADF) STEM atomic resolution imaging. To evaluate the electrons hybridized energy states and cation valence states in an SSP-TMOs nanoparticles, specifically L_2,3_ ionization edges for Mn, Fe, and Ni elements and ELNES at oxygen K-edge were analyzed. In addition, synthesized unary iron oxide (Fe_2_O_3_/Fe_3_O_4_) and manganese oxide (Mn_2_O_3_/Mn_3_O_4_) TMO nanoparticles were analyzed for chemical oxidation states and crystal structure comprehending to the chemical oxidation states evaluated from the SSP-TMOs. The possible occupancy of different metal cation sites in the localized region in the spinel crystal structure of quinary SSP-TMOs nanoparticles were evaluated using atomic resolution STEM-EELS elemental mapping. This work reveals the key attributes of chemical oxidation states associated with different metal cations in the spinel phase of successive unary, binary, ternary, and quinary SSP-TMOs nanoparticles revealing the electronic stability of quinary SSP-TMOs.

## Results

### Microstructural analysis of synthesized solid solution polyelemental transition metal oxide nanoparticles

**Figure 1:**
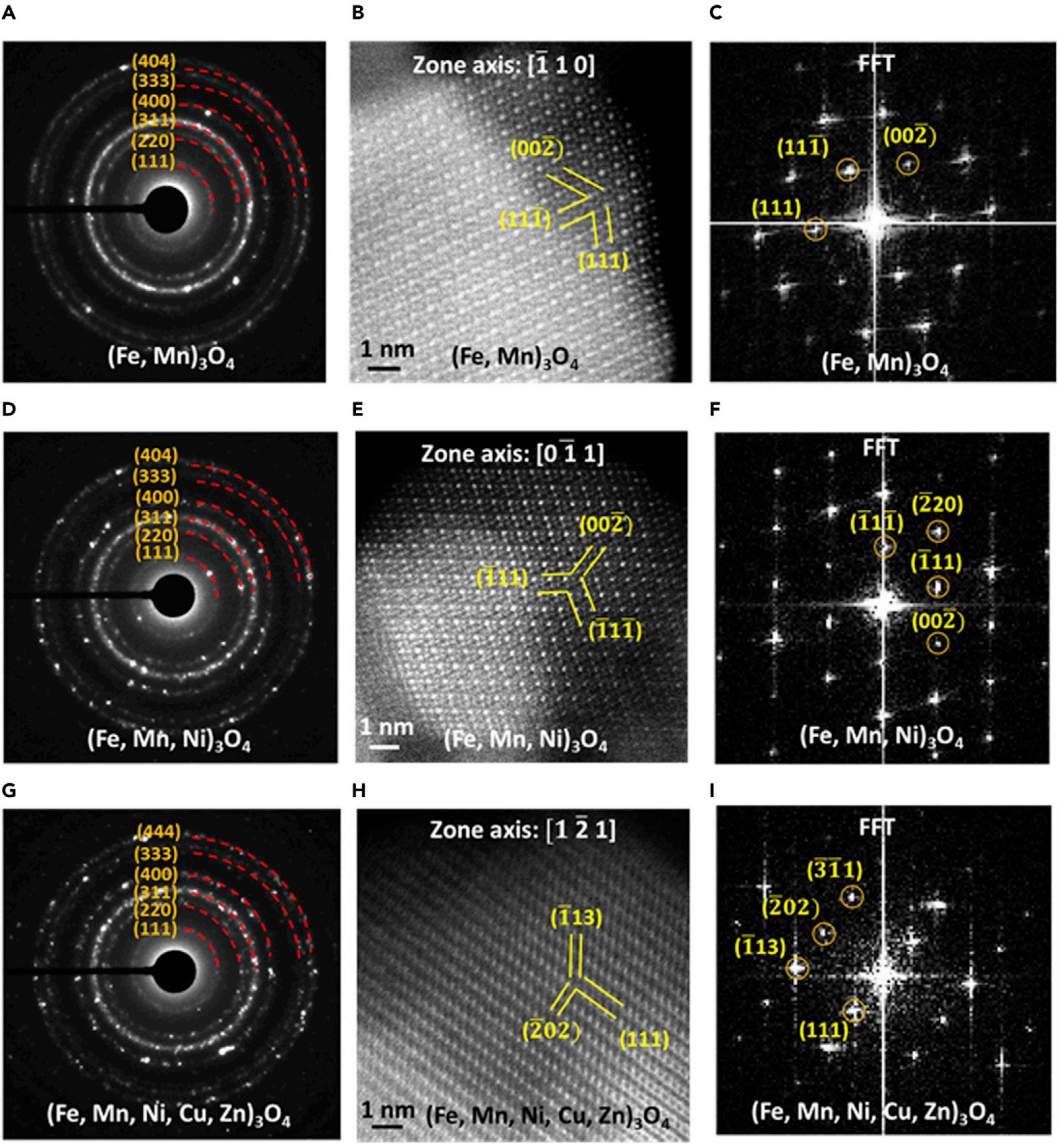
Microstructure analysis of synthesized SSP-TMOs nanoparticles (A) SAED pattern acquired from binary metal oxide (Mn, Fe)_3_O_4_ nanoparticles representing the characteristic planes of spinel crystal phase. (B) Atomic resolution HAADF-STEM micrograph of binary metal oxide (Mn, Fe)_3_O_4_ nanoparticle acquired at [$\bar{1}1$0] zone axis. Scale bar represents 1 nm. (C) Corresponding fast Fourier transform (FFT) pattern indicating the ($\bar{1}$1$\bar{1}$), (00$\bar{2}$), and (111) lattice planes in the reciprocal space. (D) SAED pattern acquired from ternary (Mn, Fe, Ni)_3_O_4_ nanoparticles representing the characteristic planes of spinel crystal phase. (E) Atomic resolution HAADF-STEM micrograph of ternary metal oxide (Mn, Fe, Ni)_3_O_4_ nanoparticle acquired at [0$\bar{1}1$] zone axis. Scale bar represents 1 nm. (F) Corresponding fast Fourier transform (FFT) pattern indicating the ($\bar{1}$1$\bar{1}$), ($\bar{2}20$), ($\bar{1}$11), and (00$\bar{2}$) lattice planes in the reciprocal space. (G) SAED pattern acquired from quinary (Mn, Fe, Ni, Cu, Zn)_3_O_4_ nanoparticles representing the characteristic planes of spinel crystal phase. (H) Atomic resolution HAADF-STEM micrograph of quinary metal oxide (Mn, Fe, Ni, Cu, Zn)_3_O_4_ nanoparticle acquired at [$1\bar{2}1$] zone axis. Scale bar represents 1 nm. (I) Corresponding fast Fourier transform (FFT) pattern indicating the ($\bar{3}\bar{1}$1), ($\bar{2}02$), ($\bar{1}$13), and (111) lattice planes in the reciprocal space.

SSP-TMOs nanoparticles were synthesized using flame spray pyrolysis route as explained in detail in the supplemental information.^4^ Spinel phase of synthesized nanoparticles was confirmed with the help of SAED analysis. Figure 1 shows the crystal structure analysis of binary (Fe, Mn)_3_O_4_, ternary (Mn, Fe, Ni)_3_O_4_, and quinary (Mn, Fe, Ni, Cu, Zn)_3_O_4_ SSP-TMOs nanoparticles. Figure 1A shows the SAED pattern acquired from the bulk quantity of binary metal oxide (Mn, Fe)_3_O_4_ nanoparticles. Spinel crystal structure characteristic diffraction rings (111), (022), (311), (400), (333), and (404) associated with respective 4.92 Å, 3.01 Å, 2.57 Å, 2.13 Å, 1.64 Å, and 1.51 Å d-spacings are represented. Figure 1B shows the atomic resolution HAADF-STEM micrograph of binary metal oxide (Mn, Fe)_3_O_4_ spinel phase nanoparticle captured at [$\bar{1}$10] zone axis. Corresponding fast Fourier transform (FFT) along [$\bar{1}$10] zone axis can be observed in Figure 1C. (11$\bar{1}$), (00$\bar{2}$), and (111) lattice planes with d-spacings 4.92 Å, 4.26 Å, and 4.92 Å are represented in the reciprocal space, respectively. Figure 1D shows the SAED pattern acquired from the bulk quantity of ternary metal oxide (Mn, Fe, Ni)_3_O_4_ nanoparticles. Spinel crystal structure characteristic diffraction rings (111), (022), (311), (400), (333), and (404) with respective 4.92 Å, 3.01 Å, 2.57 Å, 2.13 Å, 1.64 Å, and 1.51 Å d-spacings can be clearly observed, similar to the binary metal oxide nanoparticles. Figure 1E shows the atomic resolution HAADF-STEM micrograph of ternary metal oxide (Mn, Fe, Ni)_3_O_4_ spinel phase nanoparticle captured at [$0\bar{1}$1] zone axis. Corresponding fast Fourier transform (FFT) along [$0\bar{1}$1] zone axis can be observed in Figure 1F. ($\bar{1}$1$\bar{1}$), ($\bar{2}20$), ($\bar{1}$11), and (00$\bar{2}$) crystal planes with d-spacings 4.92 Å, 3.01 Å, 4.92 Å, and 4.26 Å are represented in the reciprocal space, respectively. Similarly, acquired SAED patterns from as-synthesized quinary metal oxide (Mn, Fe, Ni, Cu, Zn)_3_O_4_ nanoparticles confirming the spinel crystal structure are represented in Figure 1G. Figure 1H shows the atomic resolution HAADF-STEM micrograph of quinary metal oxide spinel phase nanoparticle captured at [1$\bar{2}$1] zone axis. Corresponding fast Fourier transform (FFT) along [1$\bar{2}$1] zone axis can be observed in Figure 1I. ($\bar{3}\bar{1}$1), ($\bar{2}02$), ($\bar{1}$13), and (111) lattice planes with d-spacings 2.57 Å, 3.01 Å, 2.57 Å, and 4.92 Å are represented in the reciprocal space, respectively. In addition, crystal structure analysis of synthesized unary iron oxide and manganese oxide nanoparticles can be referred to in Figure S1 in the supplemental information. SAED analysis of iron oxide nanoparticles as represented in Figure S1A confirms the presence characteristic lattice planes for both Fe_3_O_4_ and Fe_2_O_3_ spinel crystal phases. The atomic resolution HAADF-STEM micrograph of Fe_3_O_4_ nanoparticle is shown in Figure S1B along [001] zone axis. Corresponding FFT pattern can be observed in Figure S1C. Figure S1D represents the SAED analysis of synthesized manganese oxide nanoparticles, where the presence of characteristic lattice planes for both Mn_3_O_4_ and Mn_2_O_3_ spinel phase nanoparticles was confirmed. Figure S1E shows the atomic resolution HAADF-STEM micrograph of Mn_3_O_4_ nanoparticle along [$\bar{3}5$1] zone axis. Corresponding FFT pattern with ($\bar{1}\bar{1}$2), (103), and (211) lattice planes are shown in Figure S1F. Results indicate that the as-synthesized SSP-TMOs nanoparticles possess cubic spinel crystal phase (lattice constant 8.39 Å) with the presence of tetrahedral and octahedral cation sites. The consistent spinel crystal phase of SSP-TMOs nanoparticles make them suitable for STEM-EELS study focusing on systematic chemical oxidation state evaluation.

### STEM-EELS elemental mapping of solid solution polyelemental transition metal oxide nanoparticles

**Figure 2:**
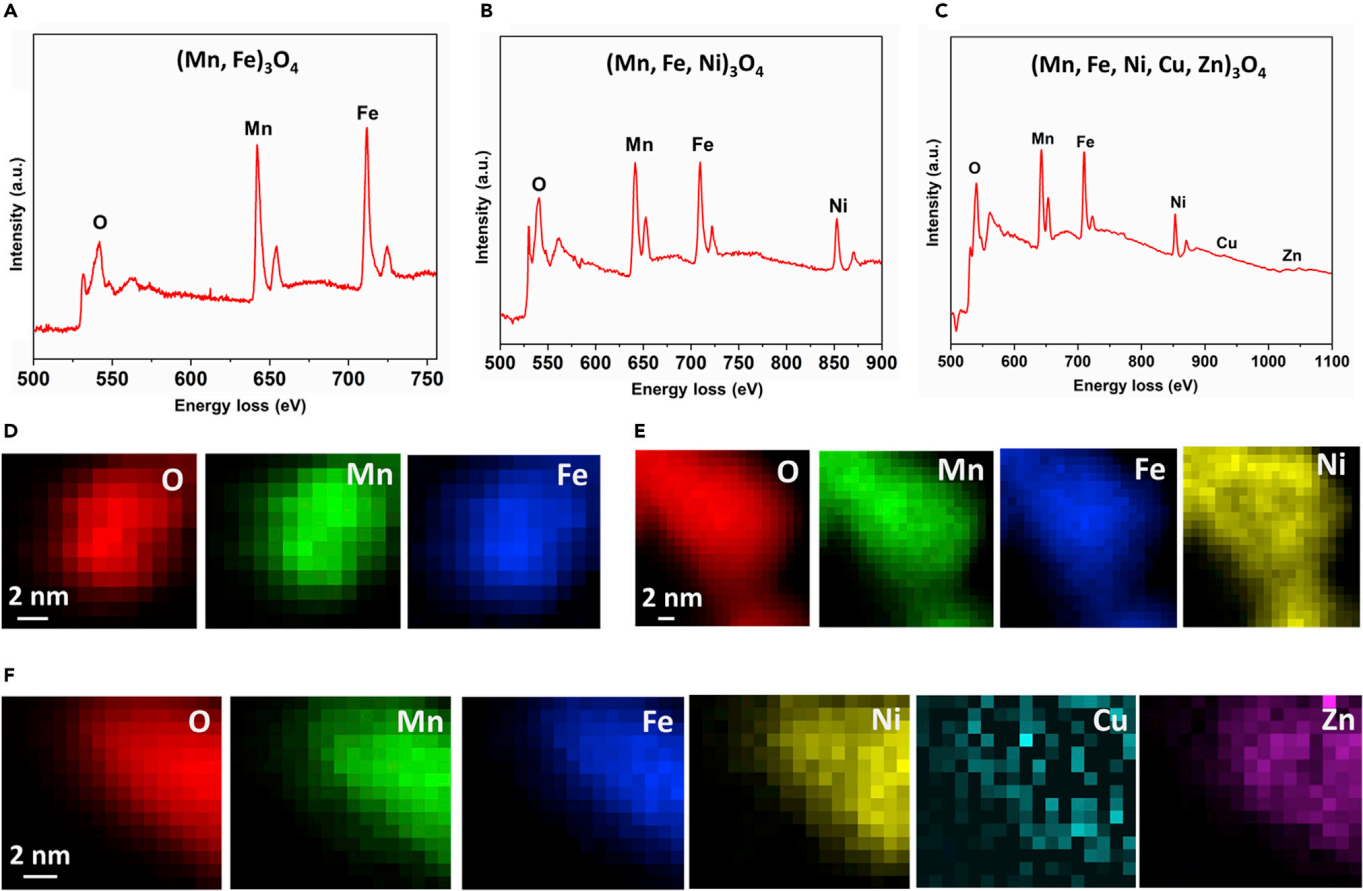
STEM-EELS elemental mapping of SSP-TMOs nanoparticles acquired from the high energy-loss L_2,3_ spectrum regions (A) EELS high energy-loss spectrum of binary metal oxide (Mn, Fe)_3_O_4_ nanoparticles acquired at 0.15 eV/Ch dispersion. (B) EELS high energy-loss spectrum of ternary metal oxide (Mn, Fe, Ni)_3_O_4_ nanoparticles acquired at 0.75 eV/Ch dispersion. (C) EELS high energy-loss spectrum of quinary (Mn, Fe, Ni, Cu, Zn)_3_O_4_ nanoparticles acquired at 0.75 eV/Ch dispersion. (D) Corresponding EELS elemental mapping of a single (Mn, Fe)_3_O_4_ nanoparticle indicating the uniform presence of oxygen, manganese, and iron elements. Scale bar represents 2 nm. (E) Corresponding EELS elemental mapping of a single (Mn, Fe, Ni)_3_O_4_ nanoparticle indicating the uniform presence of oxygen, manganese, nickel, and iron. Scale bar represents 2 nm. (F) Corresponding EELS elemental mapping of a single (Mn, Fe, Ni, Cu, Zn)_3_O_4_ nanoparticle indicating the uniform distribution of oxygen, manganese, nickel, copper, zinc, and iron. Scale bar represents 2 nm.

The elemental compositional homogeneity in binary (Fe, Mn)_3_O_4_, ternary (Mn, Fe, Ni)_3_O_4_, and quinary (Mn, Fe, Ni, Cu, Zn)_3_O_4_ SSP-TMOs nanoparticles was further evaluated using STEM-EELS elemental mapping. Figure 2 shows the high energy loss spectrum and corresponding elemental maps for spinel phase alloyed metal oxide nanoparticles. Figure 2A shows the EELS high energy loss spectrum for binary metal oxide (Fe, Mn)_3_O_4_ metal oxide nanoparticle acquired at 0.15 eV/Ch energy dispersion. The corresponding STEM-EELS elemental maps are represented in Figure 2D, where uniform distribution of iron, manganese, and oxygen elements is observed. Figure 2B shows the EELS high energy loss spectrum for ternary metal oxide (Mn, Fe, Ni)_3_O_4_ metal oxide nanoparticle acquired at 0.75 eV/Ch energy dispersion. The corresponding STEM-EELS elemental maps are represented in Figure 2E, where uniform distribution of iron, manganese, nickel, and oxygen elements is observed. Figure 2C shows the EELS high energy loss spectrum for quinary (Mn, Fe, Ni, Cu, Zn)_3_O_4_ nanoparticle acquired at 0.75 eV/Ch energy dispersion. The corresponding STEM-EELS elemental maps for manganese, iron, nickel, copper, zinc, and oxygen elements are represented in Figure 2F. The evaluated elemental composition of (Mn, Fe, Ni, Cu, Zn)_3_O_4_ quinary nanoparticles was Mn (7.63 ± 2.03) %, Fe (6.93 ± 1.18) %, Ni (7.67 ± 1.49) %, Cu (12.28 ± 4.43) %, Zn (8.21 ± 1.99) %, and O (57.82 ± 6.89) %.^4^ The detailed EELS high energy loss L_2,3_ ionization edges acquired from alloyed metal oxides at 0.15 eV/Ch dispersion with higher energy resolution are discussed in the following section. In addition, STEM-EELS high energy loss spectra with dispersion 0.15 eV/Ch and corresponding elemental mapping of unary iron oxide and manganese oxide nanoparticles are represented in Figures S1G and S1H, respectively.

### STEM-EELS and ELNES analysis of solid solution polyelemental transition metal oxide nanoparticles

**Figure 3:**
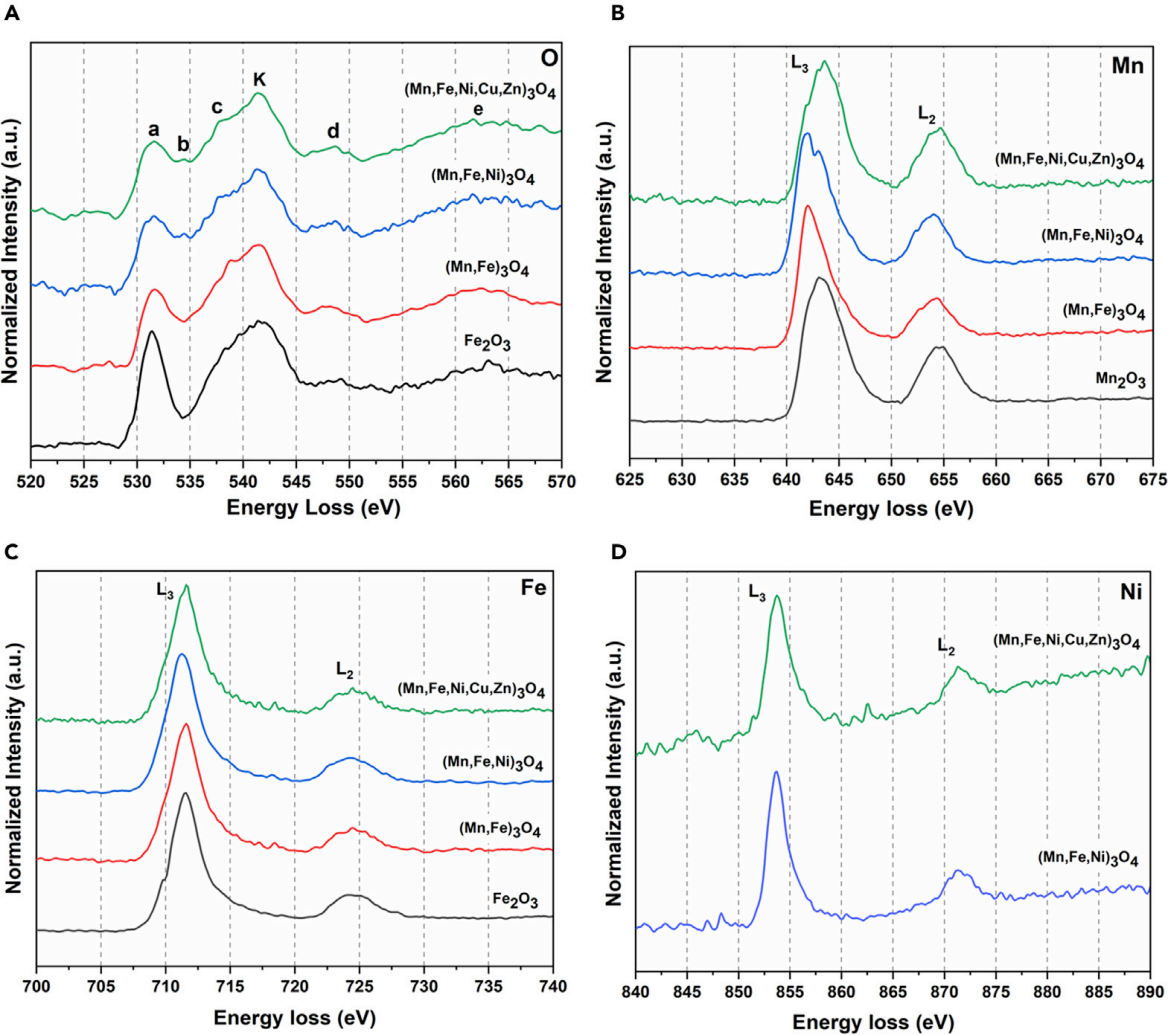
EELS and ELNES analysis for oxygen K-edge and L_2,3_ ionization edges for manganese, iron, and nickel elements in respective Fe_2_O_3_, (Mn, Fe)_3_O_4_, (Mn, Fe, Ni)_3_O_4_, and (Mn, Fe, Ni, Cu, Zn)_3_O_4_ metal oxides (A) EELS and ELNES analysis of oxygen K-edge in respective unary and SSP-TMOs nanoparticles. (B) EELS analysis of manganese L_2,3_ ionization edges in respective unary and SSP-TMOs nanoparticles. (C) EELS analysis of iron L_2,3_ ionization edges in respective unary and SSP-TMOs nanoparticles. (D) EELS analysis of nickel L_2,3_ ionization edges in respective ternary and quinary SSP-TMOs nanoparticles.

To investigate the chemical oxidation states transitioning from SSP unary to quinary metal oxide nanoparticles, systematic STEM-EELS and ELNES analysis were performed. Figure S2 shows the electron beam induced knocking damage caused to unary iron oxide (Fe_3_O_4_) nanoparticles during EELS acquisition (0.15 eV/Ch dispersion). Results confirmed more than 3-fold decrease in the intensity of oxygen K-edge pre-peak with prolonged EELS acquisition time of 99 s, where at 72 s and 87 s, the structural integrity of nanoparticles was maintained.^23^ The electron beam threshold limit was maintained during EELS acquisition assuring no influence of radiation damage to nanoparticles. The electron dose rate of 1.18 × 10^7^ e^−^/nm^2^/s was utilized during the STEM-EELS spectrum image acquisition. The oxygen K-edge and 2p core states (L_2,3_ ionization edges) for iron, manganese and nickel elements were analyzed in respective unary (Fe_2_O_3_, Mn_2_O_3_), binary metal oxide (Mn, Fe)_3_O_4_, ternary metal oxide (Mn, Fe, Ni)_3_O_4_, and quinary (Mn, Fe, Ni, Cu, Zn)_3_O_4_ nanoparticles in near equimolar elemental composition. Figure 3 represents the obtained normalized high energy loss EELS spectra for oxygen, manganese, iron, and nickel elements in respective unary, binary, ternary, and quinary SSP-TMOs nanoparticles. ELNES evolves because of the disordered distribution of the local energy levels appeared as a result of excitement of electron shells at atoms.^24^ Figure 3A shows the oxygen K-edge for unary iron oxide (Fe_2_O_3_), binary (Mn, Fe)_3_O_4_, ternary (Mn, Fe, Ni)_3_O_4_, and quinary (Mn, Fe, Ni, Cu, Zn)_3_O_4_ SSP-TMOs nanoparticles. The K-edge represented as ‘K’ appears because of the transition from oxygen 1s state orbital to 2p state orbitals. The distinct pre-edge peak prior oxygen K-edge represented as ‘a’ appears owing to the excitation of oxygen 1s orbital into empty states consisting hybridized oxygen 2p states with transition metals 3days bands.^25^ The nature of the pre-edge ‘a’ was observed to be consistent in binary metal oxide, ternary metal oxide, and quinary metal oxide nanoparticles whereas sharp oxygen pre-edge feature was observed in unary iron oxide nanoparticle. Oxygen pre-peak signifies the presence of a number of oxygen vacancies in the crystal lattice. As the number of oxygen vacancies in the lattice increases, the pre-peak intensity decreases.^26^ The results confirm that the number of oxygen vacancies is higher in quinary metal oxide nanoparticles than those present in the unary iron oxide nanoparticles. The characteristic feature ‘b’ located between oxygen pre-peak and K-edge can likely appear because of the transition into the conduction band 4p-3d hybridization of transition metal oxides.^27^ The ‘b’ feature was predominantly observed for ternary metal oxide and quinary metal oxide nanoparticles. The ELNES feature occurring post oxygen K-edge represented as ‘d’ can be attributed to the oxygen p-states mixed with transition metal 4sp band at higher energy. ELNES feature ‘e’ can appear with multiple scattering, more likely as a result of backscattering owing to oxygen shells in the complex metal oxide structures as compared with the backscattering because of transition metal cations in the indicated energy range.^28^ Both the ELNES features ‘d’ and ‘e’ showed the similar trends in all three categories of alloyed metal oxide nanoparticles. In the inverse spinel crystal structures, trivalent metal cations occupy the octahedral sites and divalent metal cations occupy either of tetrahedral or octahedral sites.^24^ The shoulder of oxygen K-edge represented as ‘c’ mainly indicates the degree of inversion among octahedral and tetrahedral sites in the spinel crystal structure.^24^ From Figure 3A, it is clearly observed that the quinary metal oxide nanoparticles possess sharper shoulder ‘c’ at oxygen K-edge in comparison with the binary metal oxide (Mn, Fe)_3_O_4_, suggesting the higher degree of inversion. In unary iron oxide nanoparticle, degree was inversion was absent. Table 1 shows white lines ratios (L_3_/L_2_) for Mn, Fe, and Ni elements of SSP-TMOs calculated from high energy loss spectrum regions represented in Figure 3. Table 1. Calculated white lines ratios (L_3_/L_2_) from high energy loss spectrum regions of Mn, Fe, and Ni elements from SSP-TMOs acquired at 0.15 eV/Ch dispersion. The corresponding Gaussian fit with coefficient of determination (R^2^) values are highlighted.

**Table 1:** Calculated white lines ratios (L_3_/L_2_) from high energy loss spectrum regions of Mn, Fe, and Ni elements from SSP-TMOs acquired at 0.15 eV/Ch dispersion

SSP-TMO nanoparticles	Manganese (Mn)		Iron (Fe)		Nickel (Ni)	
	High loss (L_3_/L_2_)	Gauss. fit (R^2^)	High loss (L_3_/L_2_)	Gauss. fit (R^2^)	High loss (L_3_/L_2_)	Gauss. fit (R^2^)
Mn_2_O_3_	2.57 ± 0.15	0.9929	–	–	–	–
Fe_2_O_3_	–	–	6.02 ± 0.21	0.9966	–	–
(Mn, Fe)_3_O_4_	3.4 ± 0.12	0.9964	5.72 ± 0.18	0.9969	–	–
(Mn, Fe, Ni)_3_O_4_	3.03 ± 0.08	0.9961	5.1 ± 0.04	0.9984	4.20 ± 0.20	0.9981
(Mn, Fe, Ni, Cu, Zn)_3_O_4_	2.44 ± 0.15	0.9929	5. ± 0.10	0.9967	4.24 ± 0.11	0.9968

The corresponding Gaussian fit with coefficient of determination (R^2^) values are highlighted.

Figure 3B shows L_2,3_ ionization edges of manganese acquired from binary metal oxide (Mn, Fe)_3_O_4_, ternary metal oxide (Mn, Fe, Ni)_3_O_4_, and quinary (Mn, Fe, Ni, Cu, Zn)_3_O_4_ nanoparticles. L_3_ edge appears owing to the transition from core state 2p_3/2_ to 3days unoccupied state, whereas L_2_ edge appears because of the transition from core state 2p_1/2_ to 3d unoccupied state. The integrated peak intensities (L_3_/L_2_) white lines ratio can be corelated to the valence energy states of transition metals.^29^ As represented in Table 1, for Mn, the calculated white lines ratios were 3.4 ± 0.12, 3.03 ± 0.08, and 2.44 ± 0.15 corresponding to the binary, ternary, and quinary SSP-TMO nanoparticles, respectively. L_3_ ionization edge can be further deconvoluted to evaluate Mn^3+^ and Mn^2+^ oxidation states contributions in the crystal structure.^30^ Deconvoluted L_3_ edge of binary metal oxides indicates the Mn^3+^:Mn^2+^ ratio as 1.61, which corresponds to characteristic ratio of nano-Mn_3_O_4_ crystals.^30^ In the ternary metal oxide nanoparticles, the calculated integrated white lines ratio is the characteristic white lines ratio of Mn_3_O_4_ nanoparticles.^29^ Deconvoluted characteristic L_3_ ionization edge of ternary metal oxides indicates the Mn^3+^:Mn^2+^ ratio as 2.74. For nano-Mn_3_O_4_ experimental Mn^3+^: Mn^2+^ ratio from deconvoluted L_3_ edge lies between 1.5 to 3.^30^ In quinary metal oxide nanoparticles, white lines (L_3_/L_2_) ratio corresponds to Mn_2_O_3_ crystal structure with +3 oxidation state of manganese.^29^^,^^31^ High energy loss STEM-EELS spectrum of quinary metal oxide nanoparticles shows that the Mn-L_3_ edge is located at 640.65 eV and Mn-L_2_ at 651.71 eV, with the energy difference (ΔE) of 11.06 eV. The STEM-EELS analysis of synthesized unary Mn_2_O_3_ nanoparticles confirms the integrated white lines ratio as 2.57 ± 0.15. Results indicate that in high entropy configuration, multiple elements can promote the stable oxidation state of manganese in the localized region in comparison with binary metal oxide nanoparticles.

Figure 3C shows L_2,3_ ionization edges of iron acquired from binary metal oxide (Mn, Fe)_3_O_4_, ternary metal oxide (Mn, Fe, Ni)_3_O_4_, and quinary metal oxide (Mn, Fe, Ni, Cu, Zn)_3_O_4_ nanoparticles. As represented in Table 1, for Fe, the calculated white lines ratios were 5.72 ± 0.18, 5.01 ± 0.04, and 5.6 ± 0.10 corresponding to the binary, ternary, and quinary SSP-TMO nanoparticles, respectively. In binary metal oxide nanoparticles, the calculated integrated white lines ratio is a fingerprint ratio of hematite (Fe_2_O_3_) nanoparticles with major Fe^3+^ oxidation state.^32^ In the ternary metal oxide nanoparticles, the calculated integrated white lines ratio is attributed to Fe_3_O_4_ spinel phase nanoparticles.^32^ Results indicate that the ternary metal oxide nanoparticles possess Fe^2+^ and Fe^3+^ oxidation states. In quinary metal oxide nanoparticles, the calculated integrated white lines ratio confirms the prominent presence of +3 iron oxidation state. STEM-EELS high energy loss spectrum of quinary metal oxide nanoparticles indicates the locations of Fe-L_3_ and Fe-L_2_ ionization edges at 709.46 eV and 722.35 eV, respectively. The energy difference (ΔE) between two ionization edges L_3_ and L_2_ is evaluated as 12.89 eV confirming the characteristic range of iron oxide.^32^ The synthesized unary Fe_2_O_3_ nanoparticles indicate that the integrated white lines ratio of iron L_3_ and L_2_ ionization edges is 6.02.

Figure 3D shows L_2,3_ ionization edges of nickel acquired from ternary metal oxide (Mn, Fe, Ni)_3_O_4_ and quinary metal oxide (Mn, Fe, Ni, Cu, Zn)_3_O_4_ nanoparticles. For standard divalent nickel oxides (NiO), the integrated white lines ratio (L_3_/L_2_) is 1.84.^33^ As represented in Table 1, for Ni, the calculated white lines ratios were 4.20 ± 0.20 and 4.24 ± 0.24 corresponding to the ternary and quinary SSP-TMO nanoparticles, respectively. In ternary metal oxide nanoparticles, the calculated integrated white lines ratio was higher than the standard +2 oxidation state of nickel. The deconvolution of Ni-L_3_ ionization edge confirms the presence of Ni^2+^ and Ni^3+^ nickel oxidation states. The nickel oxidation states (Ni^3+^: Ni^2+^) ratio evaluated from Ni-L_3_ ionization edge was 0.86 for ternary metal oxides. In the quinary metal oxide nanoparticles, Ni-L_3_ and Ni-L_2_ ionization edges lie at 851.67 eV and 869.36 eV, respectively, indicating the energy difference (ΔE) of 17.69 eV. For Ni, in quinary metal oxide nanoparticles, the calculated integrated white lines ratio was similar to ternary metal oxide nanoparticles. The ratio of nickel oxidation states (Ni^3+^: Ni^2+^) can be further evaluated by deconvoluting the Ni-L_3_ edge of quinary metal oxide nanoparticles. (Ni^3+^: Ni^2+^) nickel oxidation states ratio is observed as 0.40, suggesting the increased concentration of Ni^2+^ oxidation state in quinary metal oxide nanoparticles in comparison with that in ternary metal oxide nanoparticles.

### STEM-EELS atomic resolution mapping of entropy stabilized quinary metal oxide nanoparticle

**Figure 4:**
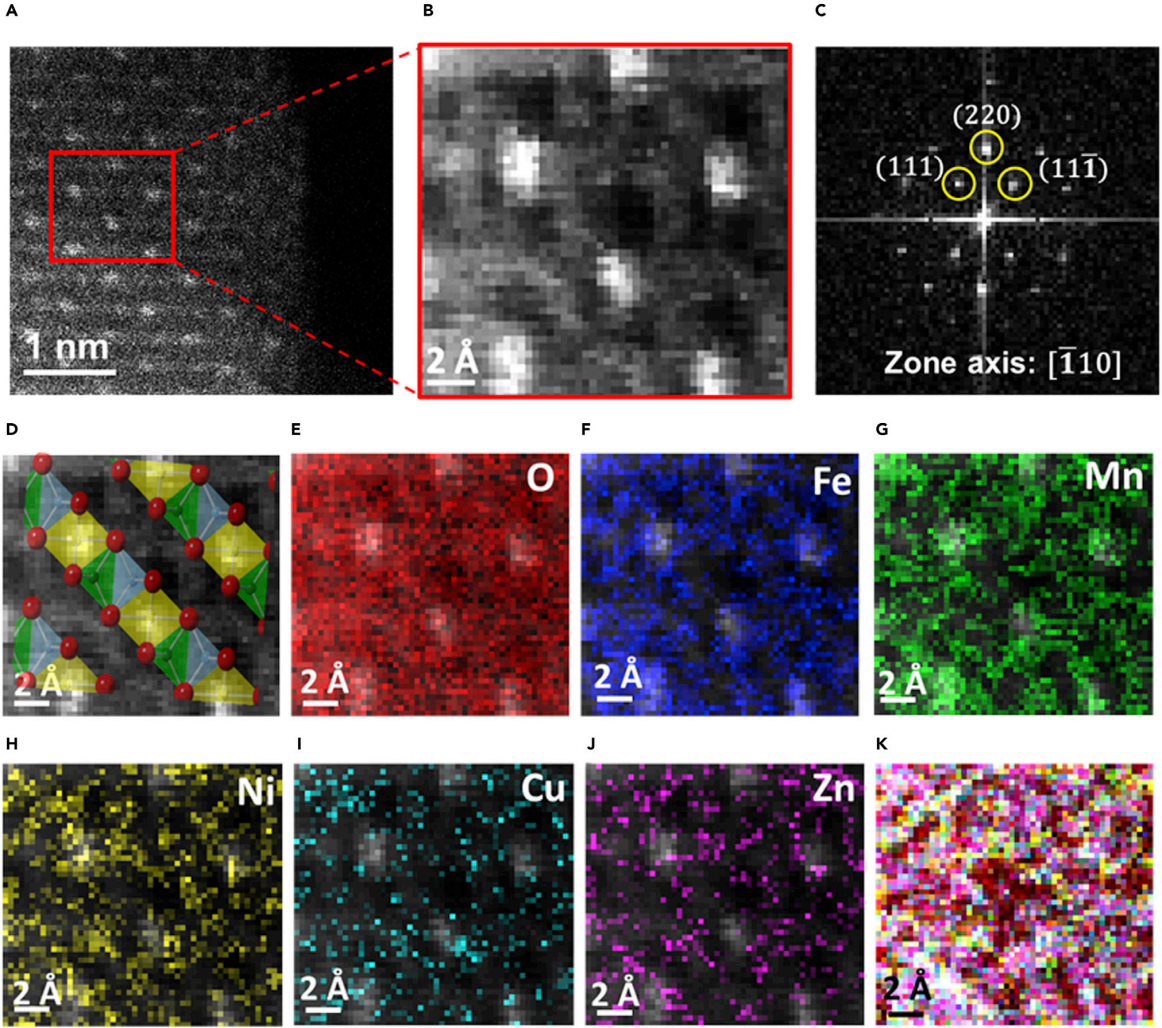
Atomic resolution EELS mapping of quinary (Mn, Fe, Ni, Cu, Zn)_3_O_4_ nanoparticle acquired at 0.75 eV/Ch dispersion (A) Annular dark field STEM micrograph of quinary metal oxide nanoparticle. Scale bar represents 1 nm. (B) Acquired EELS spectrum micrograph. Scale bar represents 2 Å. (C) Corresponding FFT analysis of EELS spectrum region indicating lattice planes in the reciprocal space along [$\bar{1}$10] zone axis. (D) Overlayed spinel phase simulated pattern at [$\bar{1}$10] zone axis confirmed the locations for octahedral sites (highlighted in yellow color), and tetrahedral sites (highlighted in green and light blue colors) occupied by metal cations. Selected oxygen atoms are represented in red color. Scale bar represents 2 Å. (E–J) Corresponding atomic resolution EELS elemental mapping of O, Fe, Mn, Ni, Cu, Zn, respectively, overlayed with EELS spectrum image. Scale bar represents 2 Å. (K) Corresponding atomic resolution EELS mixed elemental mapping of O, Fe, Mn, Ni, Cu, Zn elements overlayed with EELS spectrum image. Scale bar represents 2 Å.

Figure 4 shows the atomic resolution EELS mapping of synthesized quinary (Mn, Fe, Ni, Cu, Zn)_3_O_4_ nanoparticles. The acquisition was performed at 0.75eV/Ch dispersion to cover wide high energy loss spectrum range from O – K edge to Zn-L_3_ ionization edge. Figure 4A shows the annular dark field (ADF) atomic resolution STEM micrograph of a quinary metal oxide nanoparticle (25 Mx magnification). Figure 4B shows the STEM-EELS spectrum image (SI) acquired from the highlighted region as represented in Figure 4A. The STEM-EELS SI is acquired at 0.03 nm × 0.03 nm pixel resolution with 0.01 s/pixel acquisition time. The total acquisition time was restricted to 26 s to avoid any possible spatial drifts. Considering spatial drift challenge associated with atomic resolution imaging, there were challenges with atomic resolution STEM-EELS SI elemental acquisition. The signal to noise (SNR) ratio in the present acquired data was limited to 26 s of acquisition considering the complexity of spinel phase nanoparticles, spatial drift, and TEM vacuum column contamination. SNR during EELS acquisition can surely be improved under advanced piezo stage microscope capabilities. Figure 4C shows the FFT analysis of spinel phase quinary metal oxide nanoparticle acquired from ADF image. The FFT analysis confirmed the observed zone axis as [$\bar{1}$ 1 0] showing the orientations for (111), (220), and (11$\bar{1}$) lattice planes. Figure 4D shows the STEM-EELS SI overlapped with standard spinel iron oxide (Fe_3_O_4_) computed model highlighting the locations for octahedral sites (in yellow) and tetrahedral sites (blue and green). Computed Fe_3_O_4_ lattice model is oriented along the same [$\bar{1}$ 1 0] zone axis. Red atoms in the computed spinel phase represent the selective sites of oxygen atoms in the crystal plane. Figure 4E shows the oxygen elemental mapping in quinary metal oxide nanoparticle overlapped with atomic resolution SI. Figure 4F shows the elemental mapping of iron in quinary metal oxide nanoparticle overlapped with atomic resolution SI confirming the possible occupancy at both octahedral and tetrahedral sites in the crystal lattice. Similarly in Figures 4G and 4H respective elemental mapping of manganese and nickel overlapped with atomic resolution SI is represented. It can be observed that both manganese and nickel elements occupy both octahedral and tetrahedral sites in the lattice. Figures 4I and 4J show the respective elemental mapping of copper and zinc overlapped with atomic resolution SI. The localized region confirms that copper and zinc primarily occupied only tetrahedral sites in the crystal lattice. Figure 4K represents the mixed elemental mapping of all Fe, Mn, Ni, Cu, Zn, and O elements in the spinel phase quinary metal oxide structure confirming the high mixing entropy.

## Discussion

In summary, we studied the valance state of unary (Fe_2_O_3_ and Mn_2_O_3_), binary (Mn, Fe)_3_O_4_, ternary (Mn, Fe, Ni)_3_O_4_, and quinary (Mn, Fe, Ni, Cu, Zn)_3_O_4_ SSP-TMO nanoparticles. The microstructural SAED analysis confirms the single-phase solid solution spinel phase of synthesized nanoparticles indicating the presence of octahedral and tetrahedral cation sites. Further high-resolution STEM-EELS systematic chemical oxidation states analysis of manganese and iron metal cations in unary, binary, ternary, and quinary SSP-TMOs nanoparticles indicate the valance state of metal cations was maintained in the randomly mixed solid solution configuration. Analyzed L_2,3_ ionization edges from the high energy loss regions show that it is possible to acquire the stable oxidation states for manganese and iron elements in the quinary nanoparticles in comparison with binary and ternary metal oxides. Further oxygen K-edge ELNES features provide key insights on the increased degree of inversion among octahedral and tetrahedral sites in the quinary nanoparticles transitioning from binary and ternary metal oxides. The number of oxygen vacancies was higher in quinary metal oxide nanoparticles than those in unary iron oxide nanoparticles. In addition, the consistency with the hybridization between valence energy states of oxygen 2p state and transition metal cations 3d and 4sp higher energy states is confirmed for all binary, ternary, and quinary SSP-TMOs nanoparticles. Atomic resolution EELS mapping from the localized region indicates that the preferential occupancy of manganese, iron, nickel cations at both octahedral and tetrahedral sites. Whereas in the same localized region occupancy for copper and zinc elements was observed at only tetrahedral sites. In conclusion the study provides key insights on chemical oxidation states variation and electronic stability of metal cations in the quinary SSP-TMOs nanoparticles.

### Limitations of the study

In the present work, the atomic percentage of each element in the same category of SSP-TMOs from single nanoparticle to another will vary up to 5% considering the synthesis process limitations. It is not possible to control the concentration of multiple metal salts precursor solutions in single aerosol droplet during the ultrafast flame spray pyrolysis synthesis route before going through 1900°C flame region. STEM-EELS is highly localized and sample thickness dependent thickness; hence, the chemical oxidation state analyzed from a few nm^2^ nanoparticle region is assumed to be consistent for the whole nanoparticle.

## STAR★Methods

### Key resources table

**Table:** 

REAGENT or RESOURCE	SOURCE	IDENTIFIER
**Chemicals, peptides, and recombinant proteins**		

Manganese chloride tetrahydrate	Sigma-Aldrich	CAS #13446-34-9
Iron (III) chloride	Sigma-Aldrich	CAS # 7705-08-0
Nickel (II) chloride hexahydrate	Sigma-Aldrich	CAS # 7791-20-0
Copper (II) chloride dihydrate	Sigma-Aldrich	CAS # 10125-13-0
Zinc chloride	Sigma-Aldrich	CAS # 7646-85-7
Ethanol 200 Proof	Decon Labs	DECON LABS # 2701

**Software and algorithms**		

Digital Micrograph 3.0	GATAN	https://www.gatan.com/
OriginPro 2020	OriginLab	https://www.originlab.com/2020

### Resource availability

#### Lead contact

Further information and requests for resources and reagents should be directed to and will be fulfilled by the lead contact, Prof. Reza Shahbazian-Yassar (rsyassar@uic.edu).

#### Materials availability

All unique/stable reagents generated in this study are available from the lead contact with a completed materials transfer agreement.

### Experimental model and subject details

This work did not need any unique experimental model.

### Method details

#### Synthesis of solid solution polyelemental transition metal oxide nanoparticles

The binary (Mn, Fe)_3_O_4_, ternary (Mn, Fe, Ni)_3_O_4_, and quinary (Mn, Fe, Ni, Cu, Zn)_3_O_4_ SSP-TMOs nanoparticles were synthesized using flame spray pyrolysis route by maintaining the same process parameters. In our previous work the process parameters of flame spray pyrolysis technique can be referred illustrating the synthesis of quinary high entropy oxide nanoparticles.^4^ Briefly, the precursor solution was prepared by mixing equimolar concentration (0.01 M) of chloride metal salts in the ethanol as a solvent under constant stirring. Manganese chloride tetrahydrate (MnCl_2_·4H_2_O, Fisher Scientific), iron (III) chloride (FeCl_3_, Sigma Aldrich), nickel (II) chloride hexahydrate (NiCl_2_·6H_2_O, Sigma Aldrich), copper (II) chloride dihydrate (CuCl_2_·2H_2_O, Sigma Aldrich), and zinc chloride (ZnCl_2_, Sigma Aldrich) were used for preparing the metal salts precursor solution. Absolute ethanol (200 Proof) as solvent was procured from Fisher Scientific. For quinary metal oxide nanoparticles all five metal salts with Mn, Fe, Ni, Cu, and Zn elements were utilized. While for synthesizing binary metal oxide nanoparticles respective Mn and Fe, and for ternary metal oxide nanoparticles respective Mn, Fe, and Ni metal salts precursors were used at 0.01 M concentration. Unary iron oxide (Fe_2_O_3_/Fe_3_O_4_) and manganese oxide (Mn_2_O_3_/Mn_3_O_4_) nanoparticles were also synthesized using respective metal salts at 0.01 M concentration for acquiring the supportive STEM-EELS standard high energy loss regions. The propane torch flame with 1900°C temperature was utilized as a heating source for the synthesis.

#### Microstructural characterization of synthesized solid solution polyelemental transition metal oxide nanoparticles

To evaluate the crystal structure of binary (Mn, Fe)_3_O_4_, ternary (Mn, Fe, Ni)_3_O_4_, and quinary (Mn, Fe, Ni, Cu, Zn)_3_O_4_ SSP-TMOs nanoparticles STEM-HAADF imaging and high-resolution transmission electron microscope (HR-TEM) in the diffraction mode were utilized. To analyze the atomic resolution crystal structure of SSP-TMOs nanoparticles, aberration corrected JEOL ARM200CF TEM operated at 200 kV (at 15 μA emission current) was used. The microscope was equipped with a cold field emission gun. A convergence angle of 22 mrad was utilized for performing STEM imaging with high angle annular dark field (HAADF) detector. 8C probe size with 19 pA electron beam current was used for HAADF-STEM imaging. HAADF images of nanoparticles in Figure 1 were acquired with Orius CCD camera at 512 х 512 scanning resolution with 31.1 μS pixel dwell time. The very low electron dose rate of 31.89 e^−^/nm^2^/s was maintained during STEM-HAADF imaging. For annular dark field image in Figure 4, 15 μS of lower pixel dwell time was utilized. The SAED analysis of SSP-TMOs nanoparticles was performed by using the same aberration corrected JEOL ARM200CF microscope in the TEM mode at 200 kV. For SAED patterns acquisition diffraction mode with 25 cm camera length was utilized. Imaging conditions were maintained for all three types of metal oxide nanoparticles. Additionally, the as-synthesized unary iron oxide (Fe_2_O_3_/Fe_3_O_4_) and manganese oxide (Mn_2_O_3_/Mn_3_O_4_) were analyzed identifying their respective crystal structures using atomic resolution HAADF-STEM imaging and SAED analysis.

#### STEM-EELS elemental analysis of synthesized solid solution polyelemental transition metal oxide nanoparticles

STEM-EELS elemental analysis of synthesized binary (Mn, Fe)_3_O_4_, ternary (Mn, Fe, Ni)_3_O_4_, and quinary (Mn, Fe, Ni, Cu, Zn)_3_O_4_ SSP-TMOs nanoparticles was performed using aberration corrected JEOL ARM200CF TEM equipped with the cold field emission gun. The microscope was operated at 200 kV in the dual EELS mode. For the EELS spectrum imaging acquisition, GATAN annular dark field (ADF) detector with convergence semi-angle of 17.8 mrad and collection semi-angle of 53.4 mrad were utilized. Electron beam current 19 pA associated with 8C probe size was maintained for the EELS data acquisition. To acquire adequate EELS signal for elements with energy losses >500 eV, one should utilize high dose that may cause radiation damage especially at beam energies of 200 keV. To minimize the damage, in this work the electron dose rate did not exceed 1.18 х 10^7^ e^−^/nm^2^/s during the STEM-EELS spectrum image acquisition. The L_2,3_ ionization edges of Fe, Mn, and Ni transition metals and oxygen K-edge from respective SSP-TMOs nanoparticles were evaluated at higher resolution at 0.15 eV/Ch dispersion for chemical oxidation state analysis. To ensure the peak positions of L_2,3_ ionization edges, zero loss peak was precisely calibrated and aligned at 0 eV position. Each elemental edge was acquired with 0.15 eV/Ch dispersion at 0.87 eV full width at half maximum (FWHM) energy resolution by using aberration corrected STEM. The background signal intensity in the high energy loss region was subtracted by using power-law fitting method acquired from pre-ionization edge region using Digital Micrograph 3.0 software. Plural scattering was subtracted from the final spectra by using Fourier log method. Gaussian fits were performed in OriginPro 2020 software to evaluate white lines ratio for transitional metals in SSP-TMOs and to study their deconvoluted chemical oxidation states from L_3_ ionization edges. For acquiring EELS spectrum image (SI), the resolution of 0.8 nm × 0.8 nm was maintained with 0.3 s pixel dwell time. For the acquisition of EELS elemental mapping, energy dispersion of 0.3 eV/Ch for binary metal oxide and ternary metal oxide, and 0.75 eV/Ch for quinary nanoparticles were used. For the atomic resolution EELS elemental mapping of quinary metal oxide nanoparticle, 0.75 eV/Ch dispersion with 0.01s pixel dwell time was used. Additionally, EELS elemental mapping of unary iron oxide (Fe_2_O_3_) and manganese oxide (Mn_2_O_3_) nanoparticles were acquired at 0.15 eV/Ch energy dispersion. Further details of analysis of acquired STEM-EELS spectra are explained in the following sections.

### Quantification and statistical analysis

Figures represent averaged or representative results of multiple independent TEM/STEM experiments. Analyses and plots were performed with Digital micrograph 3.0 and OriginPro 2020 software.

### Additional resources

There are no additional resources needed to be declared in this manuscript, additional requests for this can be made by contacting the lead contact.

## Data Availability

The datasets supporting the current study have not been deposited in a public repository because these datasets are being used for further ongoing research in our labs but are available from the corresponding author on reasonable request. This paper does not report any original code.
