# Supplementary material for: Nanoscale chemical and structural investigation of solid solution polyelemental transition metal oxide nanoparticles
Source: iScience. 2023 Jan 24;26(2):106032. doi: 10.1016/j.isci.2023.106032 (PMC9929587; doi:10.1016/j.isci.2023.106032)
Supplement: Document S1. Figures S1–S3 [file mmc1.pdf]

iScience, Volume *26*

Supplemental information

Nanoscale chemical and structural investigation of solid solution polyelemental transition metal oxide nanoparticles

Abhijit H. Phakatkar, Tolou Shokuhfar, and Reza Shahbazian-Yassar

Supplemental Information

Nanoscale Chemical and Structural Investigation of Solid Solution Polyelemental Transition Metal Oxide Nanoparticles

Abhijit H. Phakatkar [1], Tolou Shokuhfar [1, *], Reza Shahbazian-Yassar [2, *]

[1] Department of Biomedical Engineering, University of Illinois at Chicago, Illinois, USA.

[2] Department of Mechanical and Industrial Engineering, University of Illinois at Chicago, Illinois, USA.

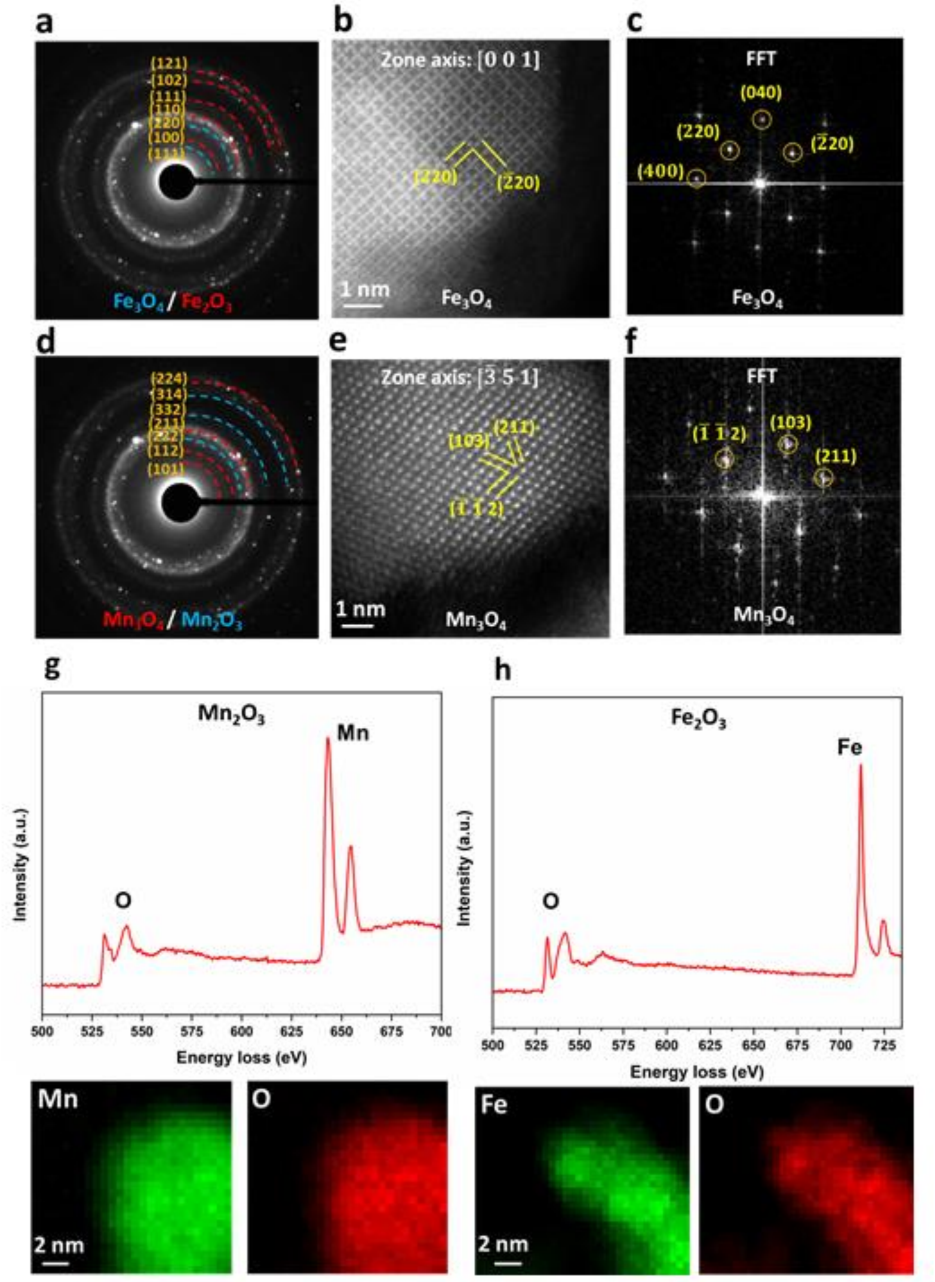

Figure S1. Crystal structure and STEM-EELS analysis of synthesized unary iron oxide (Fe_2O_3/Fe_3O_4) and manganese oxide (Mn_2O_3/Mn_3O_4) TMO nanoparticles, Related to Figure 1. (a) SAED pattern acquired from unary iron oxide (Fe_3O_4/Fe_2O_3) nanoparticles representing the characteristic planes of spinel crystal phase. (b) Atomic resolution HAADF-STEM micrograph of iron oxide Fe_3O_4 nanoparticle acquired at [001] zone axis. (c) Corresponding fast Fourier transform (FFT) pattern indicating the (040), $(\bar{2}20)$, (220), and (400) lattice planes in the

reciprocal space. (d) SAED pattern acquired from unary manganese oxide (Mn_2O_3/Mn_3O_4) nanoparticles representing the characteristic planes of spinel crystal phase. (e) Atomic resolution HAADF-STEM micrograph of Mn_3O_4 nanoparticle acquired at $[\bar{3}51]$ zone axis. (f) Corresponding fast Fourier transform (FFT) pattern indicating the $(\bar{1}\bar{1}2)$, (103), and (211) lattice planes in the reciprocal space. (g) EELS high energy-loss spectrum and associated elemental mapping of unary iron oxide (Fe_2O_3) nanoparticle acquired at 0.15 eV/Ch dispersion. (h) (g) EELS high energy-loss spectrum and associated elemental mapping of unary manganese oxide (Mn_2O_3) nanoparticle acquired at 0.15 eV/Ch dispersion.

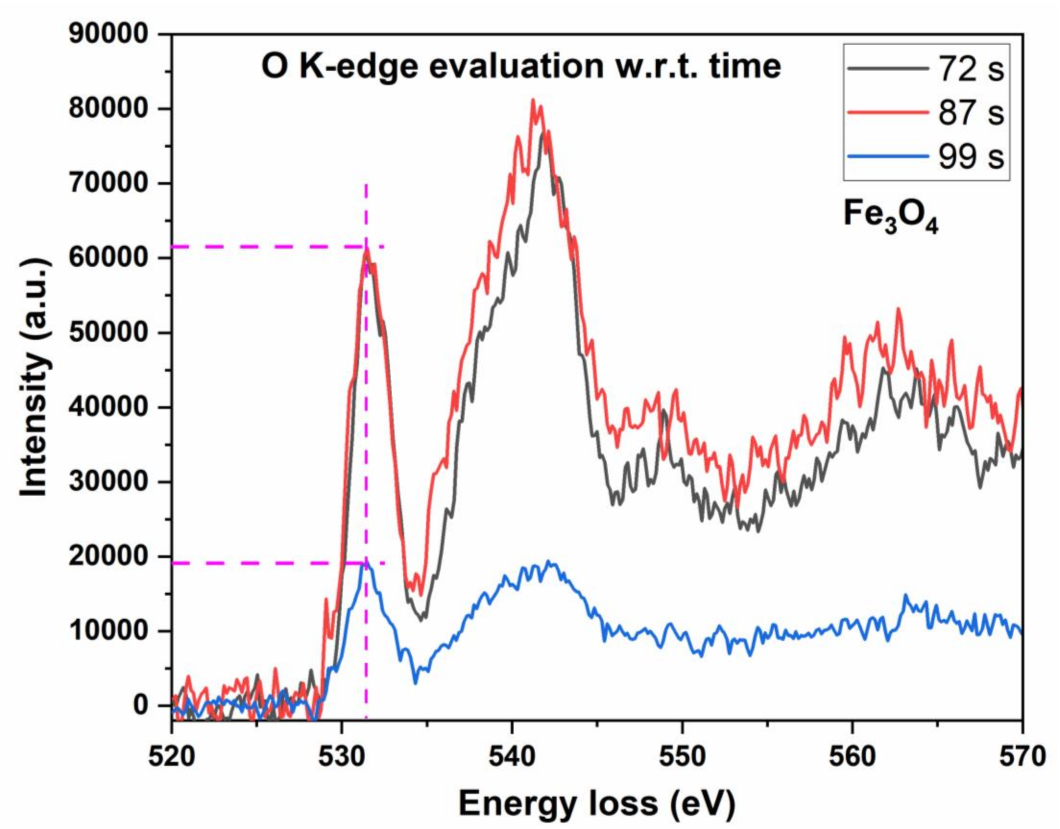

Figure S2. Electron beam induced damage evaluation performed during EELS analysis of oxygen K-edge of unary Fe_3O_4 nanoparticles, Related to STAR Methods. The relative specimen thickness

(t/λ) was maintained at 0.1. Decreased intensity of oxygen pre-peak confirms the electron beam induced knock-on damage at 99 s exposure with 19 pA beam current.
